# Saratoga Springs

**Published:** 1840

**Authors:** 


*Saratoga Springs.*—These waters have obtained a prodigious reputation in North America, and are resorted to by myriads of valetudinarians of all descriptions. We see that they are about to be imitated at Brighton, by that indefatigable chemist and mineral water manufacturer, Schweitzer.

The ingredients are potassa, soda, ammonia, lime, magnesia, strontia, protoxide of iron, ditto of manganese, alumina, silica, carbonic acid, nitric acid, sulphuric acid, iodine, bromine, chlorine. This is certainly a splendid bill of fare, but it must be remembered that the dishes are of the most homœopathic dimensions. Thus, in 1,000 grains of the water there are only about five grains and a half of the aggregate medicinal agents—scarcely enough to give the waters a taste or flavor of physic. And of these $5\frac{1}{2}$ grains, 3 are composed of soda and chlorine—nearly 1 of carbonic acid—leaving about $1\frac{1}{2}$ grains for the whole of the other constituents. We have no doubt that all true disciples of Hahnemann will flock to this spring at Brighton, and direct their patients to take, each morning, a drop of this elixir diluted in six beakers of the natural element.—*Ib.*

